# In Vitro Antigiardial Activity of Selected Plants From the Indonesian Rain Forest Identified Based on Behavioral Observations of Sumatran Orangutans

**DOI:** 10.1155/jotm/6520515

**Published:** 2026-07-30

**Authors:** Radka Pecková, Karel Doležal, Ivan Petřík, Michal Prikryl, Jana Kočířová, Wisnu Nurcahyo, Ivona Foitová

**Affiliations:** ^1^ Department of Botany and Zoology, Faculty of Science, Masaryk University, Kotlářská 2, Brno 611 37, Czech Republic, muni.cz; ^2^ Department of Chemical Biology, Faculty of Science, Palacký University, Šlechtitelů 27, Olomouc 78371, Czech Republic, upol.cz; ^3^ Laboratory of Growth Regulators, Faculty of Science, Palacký University, Šlechtitelů 27, Olomouc 78371, Czech Republic, upol.cz; ^4^ Laboratory of Growth Regulators, Institute of Experimental Botany, The Czech Academy of Sciences, Šlechtitelů 27, Olomouc 78371, Czech Republic, cas.cz; ^5^ Department of Parasitology, Faculty of Veterinary Medicine, Gadjah Mada University, Yogyakarta, Indonesia, ugm.ac.id

**Keywords:** *Giardia intestinalis*, natural antiparasitics, orangutan, parasite, plant extracts

## Abstract

**Background and Aims:**

This study evaluated in vitro antigiardial activity in 16 Indonesian plants extracted in methanol, methanol–tetrahydrofuran (1:1), and water. These plants exhibiting promising antiparasitic activity were selected on the basis of collected behavioral data and the ability of these plants to decrease parasite load in Sumatran orangutans.

**Methods:**

Plant extracts of different concentrations (0.15625–10 mg/mL) and metronidazole (100 μg/mL), a standard antigiardial drug, were incubated with 10^5^ trophozoites per milliliter of growth medium in 96‐well tissue culture plates under anaerobic conditions for 24 h. Cultures were counted in a hemocytometer using a light microscope and then statistically evaluated.

**Results:**

All tested plants were found to be effective to some extent against *Giardia intestinalis* trophozoites, with eight of them having never been previously tested for any biological activity nor probably used in ethnomedicine (as far as we know).

**Conclusion:**

Our results show that these extracts have potential as an alternative treatment of enteric diseases caused by *G. intestinalis* and confirm the assumption that orangutans use these plants for self‐medication.

## 1. Introduction

*Giardia intestinalis* is a flagellated parasitic protozoan of mammalian species, including humans. Its distribution is global, and symptomatic infections occur in both developing and developed countries [1]. In the developed world, it is considered the most prevalent parasitic cause of diarrhea [2] although infections with *G. intestinalis* are mostly associated with outbreaks [3]. Between 2004 and 2016, it caused 212 outbreaks all over the world [4, 5]. The parasite exists in two forms: trophozoites and cysts. Trophozoites colonize the upper small intestine, bile duct, or gall bladder [1, 6] and after encystation transform into a dormant cysts that are excreted in the stool and enable them to survive outside the host and invade other hosts by the fecal–oral route. Clinical symptoms of human infection include nausea, vomiting, anorexia, malaise, steatorrhea, diarrhea, epigastric pain, bloating, and malabsorption [7]. Giardiasis can also lead to long‐term changes to gut physiology, which can lead to postinfectious sequellae and predispose sufferers towards chronic gastrointestinal disorders, including irritable bowel syndrome and food allergies, which last even 10 years after parasite clearance [8].

The most common treatments against human giardiasis are 5‐nitroimidazoles such as metronidazole and quinacrine and furazolidone. This therapy is effective but can cause significant undesirable side effects [9, 10], and metronidazole was found to be carcinogenic in rodents [11]. In addition, resistance to nitroimidazoles has been recorded, reaching up to 30%–48%, especially in travelers to the tropics, and has been steadily increasing over the past decades [12, 13]. These facts are the main reasons for exploring alternative treatment strategies.

There are also alternative approaches for the treatment of giardiasis based on traditional medicine. Plants and their chemically diverse biologically active compounds represent a rich source for the development of new drugs [14]. Several naturally occurring compounds or even crude extracts from plants frequently used in ethnopharmacology to treat gastrointestinal symptoms were shown to be active against both trophozoites and cysts in vitro—for example, garlic extract caused a loss of flagellar movement and cell motility, detachment of organisms from the reaction vessel wall, and loss of osmoregularity, resulting in cell swelling and collapse of the electrochemical membrane potential [15] and ginger extract had a significant effect on cyst viability [16]. In in vivo studies, yucca extracts were shown to kill *Giardia* trophozoites in gerbils [17], while extracts from *Senna racemosa* bark were similarly effective in mice [18]. Moo‐Puc [19] tested antigiardial activity of several compounds previously isolated from *S. racemosa*. The greatest activity showed cassine, a piperidine alkaloid. Since cassine itself was almost as active as the leaves and bark extracts, the authors suggest that their activity is due in part by the presence of this alkaloid. The mechanism of action of cassine itself has not yet been tested on *G. intestinalis*, but similar study on *Leishmania major* suggests that its antiprotozoal activity could act via the ability to bind to ergosterol that constitutes the membrane of *Leishmania* [20]. It was also reported that piperine, a compound related to cassine, affects the cellular cycle od *L. amazonensis* due to significant mitochondrial alterations [21] or by interfering into the parasite lipid metabolism [22].

The plants used in this study were selected on the basis of collected behavioral data (especially feeding behavior) and the ability of these plants to reduce parasite burden in Sumatran orangutans (fecal examination). Because of the 97% phylogenetic similarity between humans and orangutans [23, 24], plants beneficial for orangutans are assumed to be promising sources for possible new treatments in human medicine. There are only a few studies dealing with the parasites of orangutans in their natural habitat and most information comes from the 21^st^ century [25–28]. Also, molecular analyses of the parasites of orangutans are rare [28]. *Giardia* spp. were first detected in wild orangutans by Mynářová et al. [29] in 2016. Although self‐medication in orangutans was first described in 1978 [30], there are only a few studies that have attempted to confirm this phenomenon [31–34].

The aim of this study was to test the hypothesis that plant extracts from selected plants in the orangutan diet exhibit antigiardial activity in in vitro conditions, which could lead to the discovery of new antigiardial treatment that would address the increasing resistance to current commercial drugs. Leaf extracts from 16 plants in three different extracting agents (water, methanol (MeOH), and methanol–tetrahydrofuran (MeOH:THF) (1:1)) were used in order to isolate the broadest possible spectrum of the compounds (both hydrophilic and lipophilic). Tested plants form a diverse group—they belong to 12 families and include both herbs, shrubs, trees, and lianas. Some plants are traditionally used in ethnomedicine to treat various disorders, some have known chemical composition, and some have been scientifically confirmed to have biological activity of various kind. However, there are several plants among them that are not used in traditional medicine (according to available information), nor do they have any confirmed biological effects, although they are widely used by local people, e.g., as a source for basketry or furniture and timber production (*R. multiflora*, *Korthalsia* spp., and *Q. argentata*) or as food (*B. scortechinii* and *E. elatior*).

## 2. Materials and Methods

### 2.1. Plant Collection

**FIGURE 1:**
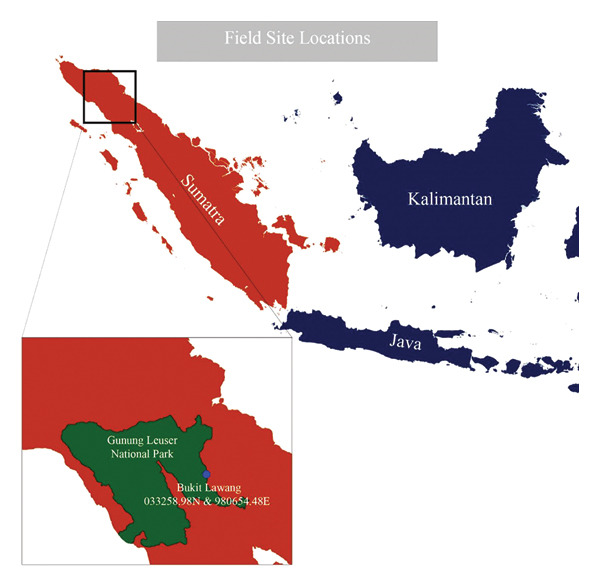
Locality of plant collection.

Tested plants (Table 1) were selected on the basis of collected behavioral data (feeding behavior). Individual orangutans were followed continuously from dawn to dusk for a period of 5 days to collect data on their daily time‐activity budgets. All types of ingested food were reported: fruit, leaves, epiphytes, flowers, bark, fungi, insects, honey, soil, etc. Fecal samples were collected regularly by taking multiple samples from individuals over different times of the day and information regarding the feces was documented. Immediately after collections, fecal samples were fixed and then examined by the standard coprological method. We found out that orangutans consume a greater quantity of certain types of plants when they are infected with parasites. Leaves of these selected plants were collected in 2008, nearby the Bukit Lawang area (the former site of a rehabilitation center for Sumatran orangutans), on the southwest border of the Gunung Leuser National Park, North Sumatra, Indonesia (N 03°32.5898′ E 098°06.5448′, Figure 1), at an altitude of 323 m above the sea level. This area is characterized by humid equatorial tropical rainforest conditions, representing mixture forest of lowland Dipterocarpaceae. Temperature in this region ranges between 21°C and 28°C, with humidity levels ranging between 70% and 90%. Annual rainfall ranges from 2000 to 3200 mm. Plants were then air dried and lyophilized. Drying of plant material was carried out over a heat source (furnace) to overcome the limitations of the high relative humidity in the tropical environment near Gunung Leuser National Park. Ambient air drying is ineffective in this region due to consistently high relative humidity (80%–90%), which reduces the vapor pressure gradient and slows evaporation. The furnace provided radiant heat, which increased the temperature of the material and accelerated water evaporation, while also enhancing convective airflow and improving moisture removal. The research was conducted in compliance with the legal requirements for undertaking research in Indonesia. A research permit was issued by RISTEK Kementarian Risetdan Teknologi. The permission to collect plant samples was obtained from LIPI (Lembaga Ilmu Pengetahuan Indonesia, Indonesian Institute of Sciences) and KKH (Kementerian Kehutanan Direktorat Jenderal Perlindungan Hutan dan Konservasi Alam). Plant identification was performed at the Botany Department, LIPI, Bogor, Indonesian Academy of Sciences by the team of Prof. Dr. Eko Baroto Walujo. The nomenclature of all plants has been updated according to Plants of the World Online (POWO) in September 2025. To contextualize these animal‐selected plants with existing scientific knowledge, Table 2 summarizes their previously reported uses in traditional human medicine and known pharmacological activities. Voucher specimens of the tested plants were deposited at the herbarium “BRNU” at the Department of Botany and Zoology of Masaryk University, Brno, Czech Republic, under individual numbers: BRNU 699,514 (*B. scortechinii*), BRNU 699,515 (*C. ornatus*), BRNU 699,516 (*C. orchioides*), BRNU 699,517 (*E. elatior*), BRNU 699, 518 (*K. laurina*), BRNU 699,519 (*K. echinometra*), BRNU 699,520 (*K. rigida*), BRNU 699,521 (*K. rostrata*), BRNU 699,522 (*M. diplotricha*), BRNU 699,523 (*P. aduncum*), BRNU 699,530 (*P. androgynus*), BRNU 699,524 (*P. pisocarpa*), BRNU 699,525 (*P. dicoccos*), BRNU 699,526 (*Q. argentata*), BRNU 699,527 (*R. multiflora*), and BRNU 699,529 (*R. anguifera*).

**TABLE 1:** Plants tested for antigiardial activity.

Botanical name	Local name	Family
*Barringtonia scortechinii* King	Medang	Lecythidaceae
*Calamus ornatus* Blume	Rotan batu	Arecaceae
*Curculigo orchioides* Gaertn.	Singgkut	Hypoxidaceae
*Etlingera elatior* (Jack) R.M. Sm.	Combang	Zingiberaceae
*Knema laurina* (Blume) Warb.	Darah darah	Myristicaceae
*Korthalsia echinometra* Becc.	Rotan kikisan	Arecaceae
*Korthalsia rigida* Blume	Rotan cincin	Arecaceae
*Korthalsia rostrata* Blume	Rotan semut	Arecaceae
*Mimosa diplotricha* C. Wright	Akar serit	Fabaceae
*Phyllanthus androgynus* (L.) Chakrab. and N.P.Balakr.	Nasi nasi	Phyllanthaceae
*Piper aduncum* L.	Sirih sirih	Piperaceae
*Popowia pisocarpa* (Blume) Endl. ex Walp.	Banitam	Annonaceae
*Psydrax dicoccos* Gaertn.	Cengkeh	Rubiaceae
*Quercus argentata* Korth	Kecing	Fagaceae
*Richetia multilfora* (Burck) P.S.Ashto and J.Heck	Semantok	Dipterocarpaceae
*Rinorea anguifera* (Lour.) Kuntze	Rambutam ayam	Violaceae

**TABLE 2:** Published information about tested plants.

Plant	Known phytochemicals	Traditional medicine	Proven effects	References
*Barringtonia scortechinii*	Alkaloids, saponins, steroids, phenols (including flavonoids), terpenoids	—	—	[35]
*Calamus ornatus*	Saponins	Treatment of canker sores and stomach ache	Antimicrobial, anti‐inflammatory, inhibitory activity against tumor cell proliferation	[36, 37]
*Curculigo orchioides*	Glycosides, lignans, polysaccharides, alkaloids, saponins, triterpenes, aliphatic compounds	Treatment of impotence, limb limpness, arthritis of the lumbar and knee joints, bronchitis, chronic cough, asthma, watery diarrhea	Antioxidant, antimicrobial, anti‐inflammatory, neuroprotective, hepatoprotective, antiasthmatic	[38, 39]
*Etlingera elatior*	Phenols (including flavonoids), terpenoids, essential oils, saponins, tannins, carbohydrates	Treatment of earache, wound cleaning	Antibacterial, hepatoprotective, anti‐inflammatory, and antioxidant activities	[40–42]
*Knema laurina*	—	Treatment of digestive and inflammatory diseases	Neuroprotective, anti‐inflammatory	[43]
*Korthalsia echinometra*	—	—	—	
*Korthalsia rigida*	Free fatty acids, glycerides, sterol esters, tannins, pholobaphenes	—	—	[44]
*Korthalsia rostrata*	—	—	—	
*Mimosa diplotricha*	Alkaloids, carbohydrates, saponins, phytosterols, phenols (including flavonoids)	Anticonvulsant and antidiabetic agent, treatment of urinary tract infections, fever, jaundice, inflammation	Antioxidant, cytotoxic, antimicrobial, antinociceptive	[45–48]
*Phyllanthus androgynus*	Lignans, nucleosides, terpenoids, flavonoids, steroids	Treatment of genitourinary, cardiovascular, gastrointestinal, and skin diseases, body weight reduction, vision improvement, lactation increasing	Anti‐inflammatory, antioxidant, immunomodulatory, antiobesity, antiulcer, antidiabetes	[49–56]
*Piper aduncum*	Flavonoids, monoterpenes, sesquiterpenes, chalcones, benzoic acid derivates	Treatment of diarrhea, dysentery, stomach ache, wounds	Antifungal, antibacterial, insecticidal, anticancer, antiprotozoal, antihelminthic, acaricidal	[57–68]
*Popowia pisocarpa*	Alkaloids, flavonoids, terpenoids	—	Antioxidant, antimicrobial	[69, 70]
*Psydrax dicoccos*	Triterpenoids, sugars, flavonoids, tannins, amino acids, sterols, carbohydrates	Treatment of fever, joint and rheumatic pains, diarrhea, inflammations	Antioxidant, antimicrobial, anti‐inflammatory	[71–73]
*Quercus argentata*	—	—	—	
*Richetia multilfora*	—	—	—	
*Rinorea anguifera*	Ellagic acids, alkaloids, glycosinolates, flavonoids	Antioxidant, antimalarial, anti‐inflammatory	—	[74]

### 2.2. Plant Extract Preparation

Lyophilized plant material was homogenized into a fine powder in liquid nitrogen and portions of the ground material (0.33 g) were extracted separately in 10 mL of water, methanol, or methanol–THF (MeOH:THF; 1:1). The following steps were performed according to the protocol of Madlener et al. [75]. Then, freeze‐dried plant extracts were dissolved in pure dimethyl sulfoxide (DMSO) and diluted with culture medium to obtain concentrations of plant extracts of 0.15625, 0.390,625, 0.625, 2.5, 6.25, and 10 mg/mL and, at the same time, a maximum concentration of DMSO of 0.06% (v/v).

### 2.3. *G. intestinalis* Trophozoites

The trophozoites of *G. intestinalis* WB isolate (ATCC 30,957) were originally obtained from duodenal aspirate of a 30‐year‐old human male with prolonged symptomatic giardiasis, probably acquired in Afghanistan, in 1982 [76], axenized and propagated in vitro in TYI‐S‐33 medium under anaerobic conditions at 37°C [77]. Since then, this strain has been maintained in the laboratories.

### 2.4. Antigiardial Activity Assay

In vitro testing against *G. intestinalis* was performed on each of the target extracts dissolved in DMSO following modified protocols of various laboratories [78–80]. A 96‐well culture plate containing culture medium was inoculated with *G. intestinalis* trophozoites to achieve an inoculum of 10^5^ trophozoites/mL. This was achieved by counting the number of trophozoites in 1 mL using a hemocytometer and then 30,000 of trophozoites was added to each 300‐μL well. Afterward, the extracts were added to the wells to obtain the required range of concentrations: 0.15625–10 mg/mL. The tested concentrations were determined based on our preliminary trials with these plants with the aim of creating the most suitable scale for calculation of IC_50_. The final volume of the fluid in each well was 300 μL. Each test included 100‐μg/mL metronidazole (a standard giardicidal drug) [80], a control (culture medium plus trophozoites and 0.06% (v/v) DMSO) and a blank (culture medium with trophozoites). After incubation for 24 h at 37°C in anaerobic conditions, trophozoites were detached by chilling and counted with a hemocytometer. The criterion used for the viability of trophozoites was their motility. The results were calculated as the percentage of live trophozoites compared to the controls grown without the plant extracts. Each concentration was tested in triplicate in two experiments.

### 2.5. Statistical Analysis

The number of living *Giardia* trophozoites counted in replicated samples (*n* = 6) was analyzed as a function of plant extract concentration. Because the response variable represented count data, i.e., nonnegative integer values, the concentration–response relationship was evaluated using negative binomial regression with a logarithmic link function. For each extract, the expected number of living trophozoites was first modeled using the following linear form:

(1) lnμ=a+bx,

where *μ* is the expected number of living trophozoites, *x* is the concentration of plant extract, *a* represents the expected log‐count in the untreated control, and *b* describes the effect of increasing extract concentration. If the decrease in trophozoite number was not adequately described by the linear model, a quadratic model was used as follows:

(2) lnμ=a+bx+cx2,

where *c* allows curvature in the concentration–response relationship.

The IC_50_ value was defined as the concentration of plant extract at which the expected number of living trophozoites decreased to 50% of the untreated control. For the linear model, IC_50_ was calculated as follows:

(3) IC50=ln0.5  b.

For the quadratic model, IC_50_ was calculated from the corresponding quadratic equation, and only biologically meaningful positive values within the tested concentration range were considered valid.

(4) IC50=−b±b2+4c ln  0.52c.

To avoid arbitrary model selection, the same decision rule was applied to all extracts: the linear model was fitted first, and the quadratic model was used only when residual diagnostics showed systematic deviation from the log‐linear fit. The detailed derivation of the IC_50_ equations is provided in the Supporting Information. The uncertainty of IC_50_ was estimated using Monte Carlo simulation with 100,000 iterations. In each iteration, model parameters were sampled according to their estimated uncertainty, and IC_50_ was recalculated. The resulting distribution of IC_50_ values was used to describe the uncertainty of the IC_50_ estimate.

The activity of each extract was then evaluated based on the resulting IC_50_ value. The significance of concentration effects was evaluated using two‐sided *z*‐tests based on the estimated regression coefficients. Effects were considered statistically significant at *p* < 0.05. The uncertainty of IC_50_ was estimated using Monte Carlo simulation with 100,000 iterations, and the 2.5th and 97.5th percentiles of the simulated IC_50_ distribution were reported as the 95% uncertainty interval. All statistical analyses were performed in RStudio v2023.06.0, Build 421 (Posit Software PBC, Boston, MA, USA), using R v4.3.0 (R Core Team, Vienna, Austria) with the following packages: MASS v4.3.2 and ggplot2 v4.0.2.

## 3. Results

### 3.1. Antigiardial Activity Assay

**FIGURE 2:**
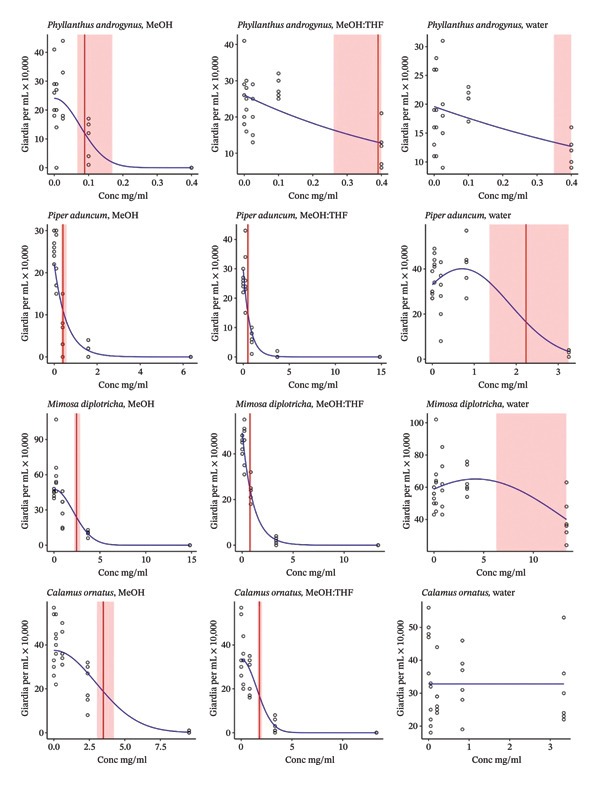
Concentration‐dependent effect of *P. androgynus*, *P. aduncum*, *M. diplotricha*, and *C. ornatus* plant extracts on the number of living *G. intestinalis* trophozoites after 24 h of incubation. Circles represent observed trophozoite counts at increasing extract concentrations, with six replicates per concentration. The blue curve shows the negative binomial regression fit with a log‐link function. The vertical red line indicates the IC_50_ estimated from the regression model, and the light red shaded region represents the uncertainty of the IC_50_ estimate based on Monte Carlo simulation. Decreasing curves reflect concentration‐dependent inhibition of trophozoite survival, while flatter curves indicate lower antigiardial activity. Blue curves with no apparent concentration‐dependent decrease indicate no detectable antigiardial activity within the tested concentration range.

Sixteen Indonesian leaf extracts in three different extracting agents (water, MeOH, or MeOH:THF) were used in this study. The antigiardial efficacy of the extracts is shown in Table 3. All the tested plants showed some activity against *G. intestinalis* in at least one extraction solvent although some of the plants were much more effective than others. The highest antigiardial effect with an IC_50_ < 1 mg/mL was achieved by the extracts of four plants: *Phyllanthus androgynus*, *Piper aduncum*, *Mimosa diplotricha*, and *Calamus ornatus* (Table 3, Figure 2).

**TABLE 3:** Estimated half‐maximal inhibitory concentration (IC_50_) values, regression models, and uncertainty intervals for plant extracts tested against *Giardia intestinalis* trophozoites after 24 h of incubation.

Name	IC_50_				Model type^c^	Antiparazitic activity^d^
	2.5%^a^	Median	Mean	97.5%^b^		
Methanolic extract						
* Phyllanthus androgynus*	0,07	0.09	0.10	0.17	Quadratic	?
* Piper aduncum*	0.32	0.41	0.42	0.58	Linear	✓
* Richetia multiflora*	1.31	1.78	1.84	2.77	Linear	✓
* Psydrax dicoccos*	1.99	2.28	2.30	2.74	Quadratic	✓
*Mimosa diplotricha*	2.19	2.46	2.48	2.87	Quadratic	✓
*Barringtonia scortechinii*	1.92	2.52	2.59	3.68	Linear	✓
* Etlingera elatior*	2.29	2.85	2.97	4.29	Quadratic	✓
*Korthalsia rigida*	2.63	3.18	3.26	4.34	Quadratic	✓
* Korthalsia echinometra*	3.10	3.48	3.51	4.07	Quadratic	✓
* Calamus ornatus*	3.03	3.49	3.53	4.24	Quadratic	✓
* Curculigo orchioides*	2.69	3.67	3.81	5.73	Linear	✓
* Korthalsia rostrata*	3.25	4.21	4.32	5.99	Linear	✓
* Shorea sumatrana*	4.20	4.73	4.76	5.51	Quadratic	✓
* Rinorea anguifera*	4.62	7.07	7.87	14.95	Linear	?
* Quercus argentata*	5.41	8.34	9.18	18.10	Linear	?
* Popowia pisocarpa*	7.94	14.28	19.03	53.84	Linear	?
* Knema laurina*	14.48	22.13	24.38	46.64	Linear	?
Tetrahydrofuran–methanolic extract						
* Phyllanthus androgynus*	0.26	0.39	0.43	0.78	Linear	?
* Piper aduncum*	0.40	0.50	0.51	0.68	Linear	✓
*Mimosa diplotricha*	0.65	0.76	0.76	0.90	Linear	✓
* Richetia multiflora*	0.93	1.24	1.28	1.88	Linear	✓
* Psydrax dicoccos*	1.24	1.52	1.56	2.13	Quadratic	✓
* Calamus ornatus*	1.62	1.80	1.81	2.05	Quadratic	✓
* Korthalsia rostrata*	1.44	1.95	2.02	3.00	Linear	✓
* Barringtonia scortechinii*	2.10	2.84	2.95	4.42	Linear	✓
*Korthalsia rigida*	4.55	5.11	5.15	5.95	Quadratic	✓
* Korthalsia echinometra*	4.25	6.08	6.21	8.92	Quadratic	✓
* Popowia pisocarpa*	4.57	6.82	7.38	13.44	Linear	?
* Curculigo orchioides*	4.33	6.84	7.61	16.07	Linear	?
* Quercus argentata*	6.51	7.51	7.59	9.16	Quadratic	✓
* Rinorea anguifera*	6.94	8.56	8.84	12.31	Quadratic	✓
* Shorea sumatrana*	13.80	27.32	44.00	133.98	Linear	?
* Knema laurina*	19.49	28.45	30.37	52.50	Linear	?
* Etlingera elatior*	—	—	—	—	Null	✗
Aqueous extract						
* Phyllanthus androgynus*	0.35	0.64	0.92	2.50	Linear	?
* Piper aduncum*	1.36	2.23	2.34	3.92	Quadratic	✓
*Korthalsia rigida*	3.59	4.82	4.98	7.31	Linear	✓
* Quercus argentata*	3.86	4.96	5.08	6.93	Linear	✓
* Psydrax dicoccos*	4.56	6.53	7.72	17.97	Quadratic	?
* Richetia multiflora*	3.79	7.17	10.55	31.44	Linear	?
* Korthalsia echinometra*	6.47	8.55	8.81	12.60	Linear	✓
* Shorea sumatrana*	3.59	8.65	14.63	54.27	Linear	?
* Curculigo orchioides*	6.12	11.35	16.91	47.28	Linear	?
* Barringtonia scortechinii*	5.92	11.75	3.80	58.01	Linear	?
* Mimosa diplotricha*	6.26	15.35	15.99	82.57	Quadratic	?
* Korthalsia rostrata*	11.08	16.84	18.47	34.96	Linear	?
* Popowia pisocarpa*	—	—	—	—	Null	✗
* Knema laurina*	—	—	—	—	Null	✗
* Calamus ornatus*	—	—	—	—	Null	✗
* Rinorea anguifera*	—	—	—	—	Null	✗
* Etlingera elatior*	—	—	—	—	Null	✗

^a^2.5% percentile.

^b^97.5% percentile.

^c^Model type: linear (log‐linear fit), quadratic (fit with an added quadratic concentration term), null (no significant concentration effect within the tested range).

^d^Active extracts (✓) showed a clear concentration‐dependent decrease in the number of living *G. intestinalis* trophozoites and provided a finite IC_50_ estimate with acceptable uncertainty. Inactive extracts (✗) showed no detectable concentration‐dependent decrease within the tested concentration range. Uncertain activity (?) indicates that the data suggested a concentration‐dependent effect, but the IC_50_ estimate was unstable, with an extremely wide uncertainty interval in the Monte Carlo simulation.

Metronidazole killed all trophozoites.

## 4. Discussion

The aim of this study was to test the antigiardial activity of plant extracts of selected Indonesian plants extracted in methanol, methanol–THF (1:1), and water in in vitro conditions.

As mentioned above, all the tested plants showed some activity against giardia trophozoites although some of them were much more effective than others. The highest antigiardial effect was found in the extract of *Phyllanthus androgynus*, followed closely by *Piper aduncum*, *Mimosa diplotricha*, and *Calamus ornatus*. All four were active with an IC_50_ < 1 mg/mL. All of these plants are used in traditional medicine for a wide range of ailments, three of them also for gastrointestinal problems (stomach ache and diarrhea): *P. androgynus*, *P. aduncum*, *C. ornatus* (Table 2). There are scientific papers demonstrating their diverse biological activity but only *P. aduncum* shows antiparasitic activity, namely, against *Rhipicephalus microplus* and *Schistosoma mansoni* [63, 64], and even antiprotozoal activity has been described, e. g. against *Leishmania amazonensis*, *L. braziliensis*, *L. shawi*, *Plasmodium falciparum*, *Trypanosoma brucei*, and *T. cruzi* [65–68].

Another nine tested plants showed moderate antigiardial activity (1 mg/mL < IC_50_ < 5 mg/mL) in at least one extraction solvent: *Richetia multiflora*, *Korthalsia rostrata*, *Barringtonia scortechinii*, *Curculigo orchoides*, *Korthalsia echinometra*, *Psydrax dicoccos*, *Etlingera elatior*, *Quercus argentata*, and *Korthalsia rigida*. Six of them have never been tested for any biological activity, nor are there any records of their use in traditional medicine (Table 2). Only *P. dicoccos* and *E. elatior* are used by local people for medicinal purposes, the former one also for the treatment of diarrhea.

The remaining three plants tested in this study, *Knema laurina*, *Popowia pisocarpa*, and *Rinorea anguifera*, showed antigiardial activity with an IC_50_ > 5 mg/mL. Scientific papers concerning either the biological activity or chemical constituents of these plants are scarce; only *K. laurina* is used by indigenous people of Malaysia to treat digestive and inflammatory diseases [81].

As we can see from Table 2, the tested plants, for which we know (at least partially) the composition, contain a wide spectrum of biologically active substances; some of them appear in several tested plant species and others in only one plant. The most frequent known phytochemicals in tested plants were flavonoids, terpenes and terpenoids (including steroids), alkaloids, saponins, and tannins. Many of these groups of substances have scientifically confirmed antigiardial effects.

Biologically active substances act on giardia in several possible ways: either they act directly on their cells, or they react with nutrients necessary for the proper functioning of giardia in the intestinal lumen, causing their deficiency and subsequent starvation of giardia, or they improve the host’s defense functions. One substance can act at multiple levels or through synergy with other substances.

Many substances (flavonoids, terpenes and terpenoids, alkaloids, tannins, phytosterols, and saponins) target the cell membrane of giardia, increasing its permeability. This results in either direct cell death or absorption of active substances that begin to act on the internal mechanisms of the cell. Important constituents of all eukaryotic membranes, including those of *Giardia* are proteins, phospholipids, and fatty acids. Because of its limited lipid synthesis ability, lipids in *Giardia* are acquired from the small intestine of the host, in which the trophozoites are exposed to free and conjugated fatty acids, various sterols, phospholipids, and bile acids [82]. Flavonoids destroy giardia cell structure by the integrity of the cell membrane by several mechanisms: interaction with the protein channels and denaturing the phospholipids present in the cell membrane, altering the fluidity of the cell membrane, and altering the osmotic pathways and ions of the cells [83]. Tannins may form soluble or insoluble complexes with many polymers such as proteins and polysaccharides which causes their fixation by cell membrane of giardias [84]. Like most eukaryotic cells, membrane biogenesis in giardias requires cholesterol. Because giardias are unable to synthesize cholesterol, they must, therefore, obtain this compound from the milieu of the upper small intestine [85]. Saponins interact strongly with cholesterol by forming stable, insoluble complexes, especially in cell membranes. This interaction disrupts membrane integrity and inhibits intestinal cholesterol absorption. Similar mechanism use phytosterols. They are, due to their lipophilicity, absorbed by the human intestine after incorporation into the so‐called “mixed micelles.” These micelles derive from the emulsification of dietary fats by bile salts and allow the entry of phytosterols into the enterocytes. Most of the absorbed phytosterols are immediately re‐excreted in the intestinal lumen by efflux transporters. Cholesterol is absorbed from the gut through the same pathway used by phytosterols (because of their similarity), and these two compounds compete with each other for incorporation in the mixed micelles and subsequent absorption in enterocytes. Hence, cholesterol fractional absorption declines along with increasing amount of phytosterols present in the gut [86].

The intracellular effect of bioactive substances on giardia occurs through direct action on microbial metabolism (flavonoids, alkaloids, and tannins) [83, 86, 87], interference on microtubule dynamics (terpenes and terpenoids) [88], and induction of autophagy or encystation (phytosterols) [89]. In human body, bioactive compounds help cure giardia infection through immunomodulatory and anti‐inflammatory effects (alkaloids) [90] or as in the case of tannins, astringent effect on the intestinal mucosa and modulation of the gut microbiota [87, 91].

All this information suggests that biologically active compounds from plants have a significant antigiardial effect and it is reasonable to discover new plants and their active substances. The results obtained in this study indicate that leaf extracts of all the tested plants extracted in MeOH, MeOH:THF (1:1), and/or H_2_O exhibit activity against *G. intestinalis* trophozoites. None of these plants has ever previously been tested for antigiardial activity and eight of them have never been tested for any biological activity; nor are there any records of their use in ethnomedicine. Our results are ground breaking, especially because we discovered the medicinal potential of these plants through observations of orangutan feeding behavior. If we had not associated the atypical consumption of these plants by orangutans with their parasitic diseases, we might never have discovered the antiparasitic activity of these plants.

Demonstration of the biological activity of these crude extracts is the first step towards discovering the compounds responsible for the biological activity of the plant. The next steps comprise purification by solid phase extraction (SPE) [92] and then by preparative high performance liquid chromatography (HPLC). The obtained fractions with proven biological activity are then examined by mass spectrometry and/or nuclear magnetic resonance to clarify the structure of the active compound [75]. During this process, the *in vitro* biological activity may be completed by *in vivo* experiments. Our results show the potential of these plants to be used in the prophylaxis and treatment of giardiasis and can be a basis for further research on the structure and mechanism of action of their active compounds.

## 5. Conclusions

This study evaluated the in vitro antigiardial activity of 16 Indonesian plants selected on the basis of feeding behavioral data and the ability of these plants to reduce parasite burden in Sumatran orangutans. All the plants were found to be effective against *G. intestinalis* trophozoites, eight of them having never been previously tested for any biological activity nor probably used in ethnomedicine. These results show the potential of tested plants to be used in medical and pharmacological research and can be a basis for further research of their active compounds.

**Nomenclature**
DMSO: Dimethyl sulfoxide
HPLC: High performance liquid chromatography
IC_50_
: Half maximal inhibitory concentration
SPE: Solid phase extraction
THF: Tetrahydrofuran

## Author Contributions

Radka Pecková performed the experiment, analyzed the data, and drafted the first version of the manuscript. Karel Doležal extracted plant extracts. Ivan Petřík performed statistical analysis. Michal Prikryl participated in conducting the experiment. Jana Kočířová extracted plant extracts. Wisnu Nurcahyo contributed to the field study design. Ivona Foitová, project manager and designer, coordinated and managed the field study, selected plants for testing, participated in experimental design.

## Funding

This research was supported by Grantová Agentura České Republiky ∗ GA23‐06571S and UMI–Saving of Pongidae Foundation.

## Disclosure

All authors have read, contributed to, and approved the final manuscript.

## Conflicts of Interest

The authors declare no conflicts of interest.

## Supporting Information

Additional supporting information can be found online in the Supporting Information section.

## Supporting information


**Supporting Information** Supporting Information contains the detailed description of derivation of the IC_50_ equations.

## Data Availability

The data that support the findings of this study are available in the Supporting Information of this article.
