# Supplementary material for: In Vitro Antigiardial Activity of Selected Plants From the Indonesian Rain Forest Identified Based on Behavioral Observations of Sumatran Orangutans
Source: J Trop Med. 2026 Jul 30;2026:6520515. doi: 10.1155/jotm/6520515 (PMC13421921; doi:10.1155/jotm/6520515)
Supplement: Supplementary file 1 — Supporting Information Supporting Information contains the detailed description of derivation of the IC50 equations. [file JOTM-2026-6520515-s001.docx]

**Derivation of IC_50_ equations**

The expected number of living trophozoites was modelled using negative binomial regression with a logarithmic link function. For the linear model, the expected response was expressed as:

$$\ln\left( \mu\left( x \right) \right)=a+bx$$

where *μ*(*x*) is the expected number of living trophozoites at extract concentration *x*. The expected response in the untreated control is therefore:

$$\ln\left( \mu\left( 0 \right) \right)=a$$

The IC_50_ was defined as the concentration at which the expected number of living trophozoites was reduced to 50% of the untreated control:

$$\mu\left( {IC}_{50} \right)=0.5\cdot\mu(0)$$

After logarithmic transformation:

$$\ln\left( \mu\left( {IC}_{50} \right) \right)=\ln\left( 0.5 \right)+ln(\mu\left( 0 \right))$$

Substituting the linear model gives:

$$a+b{IC}_{50}=\ln\left( 0.5 \right)+a$$

After simplification:

$$b{IC}_{50}=\ln\left( 0.5 \right)+a$$

and therefore:

$${IC}_{50}=\frac{ln(0.5)}{b}$$

For the quadratic model:

$$\ln\left( \mu\left( x \right) \right)=a+bx+cx^{2}$$

The IC_50_ condition is again:

$$\mu\left( {IC}_{50} \right)=0.5\cdot\mu(0)$$

After logarithmic transformation and substitution:

$$a+b{IC}_{50}+c{IC}_{50}^{2}=\ln\left( 0.5 \right)+a$$

After removing *a* form both sides:

$$b{IC}_{50}+c{IC}_{50}^{2}-ln(0.5)=0$$

This quadratic equation was solved as:

$${IC}_{50}=\frac{-b\pm\sqrt{b^{2}+4c ln(0.5)}}{b}$$

The biologically meaningful positive solution within the tested concentration range was used.
